# Comparative Gene Signature of (−)-Oleocanthal Formulation Treatments in Heterogeneous Triple Negative Breast Tumor Models: Oncological Therapeutic Target Insights

**DOI:** 10.3390/nu13051706

**Published:** 2021-05-18

**Authors:** Mohammed H. Qusa, Khaldoun S. Abdelwahed, Abu Bakar Siddique, Khalid A. El Sayed

**Affiliations:** School of Basic Pharmaceutical and Toxicological Sciences, College of Pharmacy, University of Louisiana at Monroe, 1800 Bienville Drive, Monroe, LA 71201, USA; qusamh@warhawks.ulm.edu (M.H.Q.); abdelwks@warhawks.ulm.edu (K.S.A.); siddique@ulm.edu (A.B.S.)

**Keywords:** (−)-oleocanthal, extra-virgin olive oil, TNBC, MMTV-PyVT, PDX, microarray

## Abstract

Triple negative breast cancer (TNBC) heterogeneity and limited therapeutic options confer its phenotypic aggressiveness. The discovery of anti-TNBC natural products with valid molecular target(s) and defined pharmacodynamic profile would facilitate their therapeutic nutraceutical use by TNBC patients. The extra-virgin olive oil (EVOO) is a key Mediterranean diet ingredient. *S*-(−)-Oleocanthal (OC) leads the bioactive anti-tumor EVOO phenolic ingredients. A previous study reported the solid dispersion formulated OC with (+)-xylitol (OC-X) suppressed the in vivo progression and recurrence of the TNBC MDA-MB-231 cells. This study investigates the ability of OC-X formulation to suppress the in vivo heterogeneous BC initiation and progression utilizing advanced preclinical transgenic MMTV-PyVT and TNBC PDX mouse models. Furthermore, the clustering of the gene expression profiles in MMTV-PyVT and PDX mouse tumors treated with OC-X acquired by a Clariom S microarray analysis identified the distinctly affected genes. Several affected novel signature genes identified in response to OC-X treatments and proved overlapped in both mouse and human tumor models, shedding some lights toward understanding the OC anticancer molecular mechanism and assisting in predicting prospective clinical outcomes. This study provides molecular and preclinical evidences of OC-X potential as a nutraceutical suppressing heterogeneous TNBC model and offers preliminary gene-level therapeutic mechanistic insights.

## 1. Introduction

According to the surveillance research of the American Cancer Society, more than 3.8 million US women with a history of breast cancer (BC) were alive in January 2021 [1,2,3]. Some of these survivors were partially or fully cured. Unfortunately, more than 150,000 BC cases are living with the metastatic disease [1].

Triple negative breast cancer (TNBC) is one of the BC subtypes defined by the lack of ER, PR, and HER2 expression, making this BC phenotype the most pathologically aggressive, with a higher risk of local and distant relapses and being more challenging to control [4,5,6]. Recently, numerous studies have highlighted the immune checkpoints as a key player in TNBC modulation and prevention [7,8]. However, the limited availability of effective targeted therapies for this type of BC phenotype is attributed to the paucity of viable molecular targets and the sophisticated tumor microenvironment. Clinical evidence also suggests that the TNBC microenvironment is associated with rapid gene mutations via the induction of several metastatic and resistance factors [9]. Although many natural dietary compounds are potent in suppressing BC growth, such as naringin, sulforaphane, and piperine, their role in preventing TNBC with clear molecular mechanisms remains largely pleotropic and unknown [10,11]. Therefore, novel therapeutic strategies and treatments with a defined pharmacodynamic profile are urgently needed to improve the treatment efficacy and control the recurrence and metastatic potential in TNBC patients and survivors.

*S*-(−)-Oleocanthal (OC), a well-known bioactive dietary phenolic, was first purified from extra virgin olive oil (*Olea europaea*) (EVOO) by Motedoro et al. in 1993 [12]. Cumulative pharmacological studies show that OC has a wide range of health benefits as a major mono-phenolic secoiridoid ingredient in EVOO, such as antioxidants, Alzheimer’s disease hallmark Aβ amyloid suppression, and anti-inflammatory and hypolipidemic effects [13,14,15]. Furthermore, OC has been reported to control a wide range of cancer pathways, including proliferation, migration, invasion, and angiogenesis among multiple cancer types [16]. OC possesses its activity through the disruption of c-Met related pathways as a prototypic member of a unique subfamily of receptor tyrosine kinases (RTKs) [17,18], while phosphorylated (activated) c-Met is considered an important predictor of tumor aggressiveness, poor survival, and metastatic potential, including TNBC [19]. Targeting the c-Met kinase domain with OC suppresses multiple downstream molecular effectors, such as PI3K, mTOR, STAT3, paxillin, Brk, FAK, HSP90, and MAPK pathways, etc., which mediates potent anticancer effects [18,20]. Nevertheless, along with increasing knowledge of OC anticancer activity, the impact of OC on the expression of genes associated with the development of TNBC is still not fully understood.

Xylitol is one of the most widely used carriers in pharmaceutical formulations among the polyols [21]. Previously, we developed a novel solid dispersion oral formulation that involved complexation between OC and (+)-xylitol (OC-X) [22]. It has been proven that OC-X can be a potential nutraceutical for effective control and prevention of the mice TNBC xenograft model in nude mice using the MDA-MB-231 cell line in three different modes: prophylactic, growth, and recurrence [22]. Results suggested the use of OC-X as a nutraceutical formulation not only to potential TNBC patients and survivors, but also to women with cancer risk factors. In the past few years, there have been numerous types of BC models that challenge formulated or plain OC bioactivity in vitro and in vivo, related to the growing appreciation of OC as a lead compound targeting c-MET for treatment and prevention of TNBC [17,18]. However, most of these BC models are single cell line-based mouse models that cannot mimic the human clinical situations in terms of heterogeneity and complexity [18,23]. Genomic heterogeneity, gene alterations, and mutations across TNBC patients became a progressive oncological research theme of high clinical interest [24]. To the best of our knowledge, there were no attempts to assess OC potency against heterogeneous TNBC models. Therefore, to develop a translational and preclinical understanding of the OC anti-TNBC pharmacodynamics at the molecular and gene levels, OC-X was selected for activity and gene signature studies in two heterogeneous TNBC models. The mammary-specific polyomavirus middle T antigen overexpression mouse model (MMTV-PyVT) can rapidly develop multifocal tumors and extensive lung metastasis, making it a common transgenic mouse model to study TNBC progression and metastasis [25]. Despite the fact that it is not primarily a human tumor, MMTV-PyVT mimics the receptor tyrosine kinase signaling pathways commonly activated in human malignancies, including TNBC, which qualifies this model as a valid heterogeneous preclinical animal model [26]. On the other hand, patient-derived xenograft (PDX) mice have recently been developed to reflect the heterogeneity of patient oncogenes [27]. PDXs maintain the clinical heterogeneity, tumor behavior, and genomic signatures of their primary tumors over multiple serial passages, providing valid pseudo-clinical models useful for reflecting the parent tumor response for various treatments [27,28]. Studying the ability of novel anticancer leads like (−)-oleocanthal to alter the genetic profile of these two advanced heterogeneous models, open the door to fully understand its molecular mechanism at the gene level.

Bioinformatics play progressively important roles in clinical oncology, therapeutic mechanisms, and target prediction [29]. In addition, using bioinformatics software such as Ingenuity Pathway Analysis (IPA) to compare the gene signature levels against a large genomics database is a widespread tool in areas such as pharmacogenomics that facilitate the identification of the key features and expression signatures for individual pathways and genes [30].

This study demonstrates the anti-TNBC effects of OC-X formulation treatments utilizing two advanced preclinical heterogeneous animal models. Oral administration of OC-X curbed the initiation and progression of TNBC in vivo in MMTV-PyVT and PDX mice. In addition, microarray analysis shed light on the ability of OC-X formulation to manipulate the gene-level profile of human-like tumors in a way that controls tumor progression and metastasis. Notably, comparison of the gene expression levels of the MMTV-PyVT and PDX mice tumors after OC-X treatments revealed affected biomarker key modulators of OC activity against TNBC. This study provides insights into the OC molecular mechanism at the gene level, which is important for its future development as a precision therapy targeting TNBC-specific molecular markers.

## 2. Materials and Methods

### 2.1. Chemicals and Reagents

All chemicals were purchased from VWR International (Suwanee, GA, USA), unless otherwise stated. *S*-(−)-Oleocanthal (OC) was extracted from EVOO (The Governor, Corfu, Greece), and a purity of >99% was established based on q^1^H NMR analysis [31]. OC extraction, purification, and analysis methods were extensively described earlier [31]. (+)-Xylitol (99% purity, No. X00810) was purchased from Pflatz and Bauer (Waterbury, CT, USA). Water used throughout the study was double distilled. All other products and reagents were of ACS or analytical grades.

### 2.2. Animal Models and Treatment Mode

All animals were acclimated at the University of Louisiana at Monroe (ULM) vivarium and maintained under clean room conditions in sterile filter top cages with Alpha-Dri bedding, and were housed on high-efficiency particulate air filtered ventilated racks at 25 °C, 55–65% relative humidity, and a 12 h light/dark cycle for a week before the experiments. The mice had free access to drinking water and pelleted rodent chow (No. 7012, Harlan/Teklad, Madison, WI, USA). All animal experiments were approved by the Institutional Animal Care and Use Committee, ULM, protocol number 17 APR-KES-03, and were handled in strict accordance with good animal practices defined by the NIH guidelines.

#### 2.2.1. Preparation of Oleocanthal-Xylitol Solid Dispersion (SD) Formulation for Animal Dosing

The (−)-Oleocanthal and (+)-xylitol (1:7) SD formulation was prepared using the hot melt fusion method as previously described [22]. Briefly, (+)-xylitol, m.p 93–100 °C, a hydrophilic carrier, was melted by using an IKARCT basic hotplate at 100 °C, and OC (5 mg per batch) was then added. The SD mixture of OC-xylitol was made to formulate OC, an oily compound, into a powder form. OC was added to melted xylitol at a ratio of 1:7, *w*/*w*. This ratio proved ideal for creating a favorable powder formulation, which has been fully characterized [22]. The molten mixture was quickly cooled by pouring into a non-sticky pan and subjected to N_2_ flow for 5 min. Once solidified, the mixture was grinded and stored at room temperature in a dark place for at least 24 h before analysis.

#### 2.2.2. Antitumor Activity of OC-X Formulation in Transgenic MMTV-PyVT Mice

Three-week-old female FVB/N-Tg (MMTV-PyVT) 634Mul/J transgenic mice were purchased from Jackson Laboratory (Bar Harbor, ME, USA). At this age, mice express the polyoma virus middle T antigen transgene at high levels in the mammary glands (10 glands/mouse) [32]. The spread of palpable mammary tumors in any gland has not yet been observed at this age. The mice were randomly divided into two groups: (1) placebo-treated control group with xylitol and sucrose (*n* = 3/30 glands); and (2) OC-X 1:7 SD formulation-treated group (*n* = 4/40 glands). Mice treatment started at the age of four weeks after appropriate accommodation via oral gavage; 6×/week for seven weeks. Animal tumor progression was monitored twice a week for total tumors present in all glands. Tumor volume (V) was deduced by: V = L/2 × W2, where L is the length and W is the tumor width. Mice body weight and general health signs, including food and water consumption, defecation, urination, and physical activity, were carefully monitored daily. All mice were sacrificed on the 56th experiment day, and all tumors were excised, collected, and weighed. Lung morphological change analysis was performed to characterize lung metastasis by lung fixation technique. Briefly, the mice thorax was opened, and blood was exsanguinated through the caudal vena cava, then, a 28-gauge needle was inserted into the trachea and secured with a chromic gut suture (Mycomedical, Apex, NC, USA). The lungs were fixed by gentle infusion in 10% formalin as a fixative reagent.

#### 2.2.3. Antitumor Activity of OC-X in a Breast Cancer Patient-Derived Xenograft (PDX) Model

Eight-week-old female non-obese diabetic/severe combined immunodeficiency gamma (NOD/SCID gamma) patient-derived xenograft (PDX) tumor-bearing mice were subcutaneously implanted with P2 and P5 fragments of a human patient-derived BC xenograft TM00089/lineage 037 purchased from Jackson Laboratory (Bar Harbor, ME, USA). The patient tumor was reported as ER positive/PR negative/ERBB2 negative in early passages lineage (039), while mutated to triple negative for lineage 037. Mice were randomly divided into two groups consisting of five mice in each group: (1) placebo-treated control group with xylitol and sucrose; and (2) OC-X 1:7 SD formulation-treated group, with an equal amount of sucrose. Treatment was started after one week of mice accommodation via oral gavage, 6×/week under sterile conditions for three consecutive weeks. Animal tumor progression was monitored every other day. Tumor volume (V) was deduced by: V = L/2 × W2, where L is the length and W is the tumor width. Mice body weight and general health signs were carefully monitored daily. All mice were sacrificed on the 21st experiment day, and individual tumors were excised and weighed.

### 2.3. RNA Extraction for Genomic and Transcriptomic Profiling

A microarray chip process was performed for MMTV-PyVT and PDX tumor specimens as previously described [20]. The total RNA was extracted from at least three samples using the TRIzol reagent (Thermo Fisher Scientific, Inc.) conducted in conformity with TRIzol/Phase Lock Gel protocol and purified with an RNeasy mini kit (Qiagen GmbH, Hilden, Germany). Quality control of the isolated RNA was performed using the Agilent TapeStation 4200 RNA ScreenTape assay kit (Agilent, 5067–5576) to determine the RNA integrity number (RIN) and concentration of the RNA; cRNA has been fragmented, and the RNA ScreenTape assay of the biotin-labeled targets performed prior sample hybridizations, then were hybridized to an Affymetrix Mice and Human Clariom S Array (Affymetrix; Thermo Fisher Scientific, Inc.) using the GeneChip system for MMTV-PyVT and PDX samples, respectively. All arrays were scanned using the Affymetrix GeneChip Command Console, which was installed in the GeneChip Scanner 3000 7G. All array datasets were analyzed using Affymetrix default analysis settings and global scaling as a normalization method and were uploaded onto the microarray data management system (MDMS) for storage. All array experiments were performed at the University of Kansas Medical Center Genomics Core Facility (Kansas City, KS, USA).

### 2.4. Data Normalization and Gene Ontology Enrichment Analysis

Array datasets were expressed as fold-change values, and differentially expressed genes were identified using Affymetrix’s Transcriptome Analysis Console (TAC) software applying the Clariom S human/mice library for comparison of the groups. Gene values were considered differentially organized between the vehicle and OC-X formulation-treated groups when there was a ≥ 1.5-fold difference in absolute expression. Differentially expressed gene fold changes in either the positive or negative direction were deemed up- or downregulated, respectively. The gene ontology (GO) biological technique was appended to assess the primary function of the differential expression of mRNAs. The GO system of classification organizes genes into hierarchical categories and uncovers the gene regulatory canonical pathways based on molecular function (e.g., catalytic and transporter activity), biological process (e.g., signal transduction and homeostasis), and cellular component (e.g., cytoplasmic and nuclear genes) [33].

### 2.5. Pathway Analysis

Ingenuity pathway analysis (IPA) software was utilized to discover the affected pathways and biomarker genes in the array datasets and to explore and correlate novel OC-X-targets with known biology responses [34]. IPA relies on computer algorithms that analyze the functional connectivity of the genes from information acquired within the IPA database [34]. Differentially expressed transcripts obtained from mouse and human Clariom S array of MMTV-PyVT and PDX mice tumor specimens, respectively, were functionally analyzed according to the QIAGEN IPA Knowledge Base (IPKB). The core analysis function feature in the software was used to interpret the differentially expressed data, which included canonical pathways, biological processes, up- and downstream transcriptional regulators, and gene networks. In addition to the tool most broadly utilized in this work, the comparison analysis, which identify trends, similarities, and differences between genes of mouse and human origin, was initialized in MMTV-PyVT and PDX mice, respectively. Each eligible gene of interest identified was then mapped to its corresponding gene functions in the IPKB. Gene network generation was applied through the IPA building tool to link specific genes of interest from datasets.

### 2.6. Statistics

Data analysis was performed using GraphPad Prism software version 8.4.3. (La Jolla, CA, USA). The results are presented as mean ± standard error of the mean (SEM) for continuous variables. Differences among various treatment and control groups in the animal study were determined by the paired *t*-test and *p*-value implication: * *p* < 0.05, ** *p* < 0.01, and *** *p* < 0.001.

## 3. Results

### 3.1. OC-X Inhibits Breast Cancer Growth and Metastasis to Lungs in MMTV-PyVT Transgenic Mice

**Figure 1:**
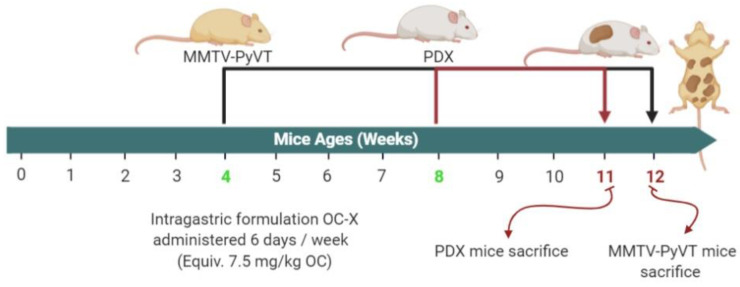
Schematic overview of in vivo studies in MMTV-PyVT and PDX mice. Mice were randomly divided into placebo control and OC-X treated groups. OC-X was dissolved in sterile water and administered by oral gavage 6×/week, at a dose equivalent to 7.5 mg/kg OC. MMTV-PyVT mice began treatments on the fourth week of age and ended at week 12, while the PDX mice began treatments on the eighth week of age and ended at week 11.

**Figure 2:**
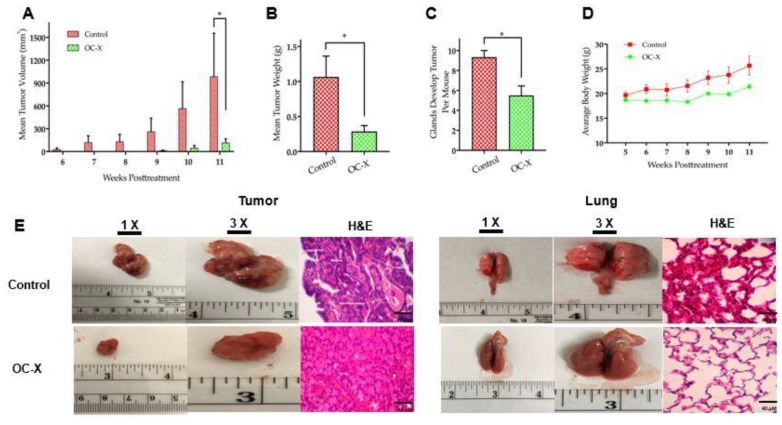
OC-X suppressed tumor growth and metastasis in the lungs in MMTV-PyVT transgenic mice. (**A**) Tumor volumes monitored for treatment and placebo control groups and measured over the 4th to 12th weeks of mice age. A caliper was used to measure individual tumors with the formula V = (W2 × L)/2, where V is the volume, W is the width, and L is the length of the tumor. The results show significant tumor suppression in OC-X treated mice (7.5 mg/kg OC by oral gavage, 6×week, *n* = 4 mice) versus the control group (*n* = 3 mice). (**B**) Comparison of the collective mean tumor weight collected from the glands of treatment and control groups after mice were sacrificed at the end of the experiment. (**C**) Comparison of the incidence of tumor development in OC-X treated versus control group mouse glands. OC-X treatments reduced the incidence of palpable lesions in most of the treated mouse glands (22 mammary glands out of 40 had tumors; 55%), leading to decreased mice tumor burden in the treatment group versus the control (28 mammary glands out of 30 had tumors; 93%). (**D**) The mean average body weights for each mice group were monitored over the experiment duration. No significant mice body weight change was observed between treated and control animals. (**E**) Comparison of representative tumor images of the OC-X treatment versus control group mice. Photographs are shown in two magnifications, 1× and 3×, highlighting the vascularity and morphological changes of mice tumors and lungs in both groups. H&E staining was utilized to compare treated and control mice tumor micromorphology using 40× magnification. *p*-value indicated by using Student’s *t*-test, * *p* < 0.05.

The encouraging in vitro and in vivo results of OC anti-TNBC activity via targeting the critical receptor tyrosine kinase (RTK) c-Met warranted further in vivo studies in more advanced heterogeneous tumor models [20,35,36,37]. The MMTV-PyVT mice are a commercially available unique genetically engineered mice model of TNBC [25]. MMTV-PyVT female mice spontaneously develop luminal-like premalignant hyperplasia mammary intraepithelial neoplastic lesions in most mammary glands by the age of three weeks, and these spread throughout the entire mammary fat pad by seven weeks age [25]. This mice model mimics the pathological processes and microenvironments found in human TNBC [25]. The tumor onset is highly accelerated, quickly reaching 1.7–2.5-fold, and mammary lesion extent and histologic grades reach peak multifocal dysplastic level by 12 weeks age [25]. MMTV-PyVT mice have a high chance of developing pulmonary metastasis, which occurs in 80–90% of mice [32]. Accordingly, investigation of OC-X activity in MMTV-PyVT mice presents an ideal preclinical model for heterogeneous mammary carcinogenesis to study OC pharmacodynamics and molecular mechanisms (Figure 1). OC-X (equivalent to 7.5 mg/kg OC, *n* = 4) and placebo (*n* = 3) were administered to mice 6×/week by oral gavage, started on the fourth week of mice age and continued for seven consecutive weeks. Mice were monitored by daily measuring of their body weight, palpable tumor initiation, and volume in each mammary gland. OC-X therapeutic effects were evaluated by comparing the incidence of palpable lesions and the volume and weight of collected tumors at the end of the experiment. OC-X formulation evidently delayed the emergence of palpable lesions, especially in the lower mammary glands of the mice body for three weeks compared to the first visible lesion observed in the control group, which appeared after six weeks of the mice’s life (Figure 2A). Moreover, tumor progression was significantly reduced by OC-X treatments compared to the control group. In particular, the observed mean tumor volume between the 6th and 11th weeks were markedly decreased in the OC-X treated group. This tumor burden reduction translated into a 90% inhibition of total tumor growth compared to the placebo control group on the last study day (Figure 2A). The mean tumor weight for the OC-X treated group was 0.28 ± 0.08 g compared to 1.06 ± 0.29 g for the placebo group, i.e., 73% inhibition of the tumor burden (Figure 2B). By the middle of the 11th week, mice were sacrificed when the tumor size of one mouse in the control group approached 2000 mm^3^, the cutoff tumor burden limit specified in the approved protocol. Nearly 28 mammary glands out of 30 (93%) developed tumors in the control-treated group, while only 22 glands out of 40 developed tumor lesions in the OC-X treated group (55%, Figure 2C). Over the course of the experiment, there was a small body weight reduction observed during the earlier study days in the OC-X treated mice group, while no other adverse effects or clinical pattern changes were observed versus the control group (Figure 2D).

The effects of OC-X treatment on the cellular morphology and histology of mice tumors were examined using hematoxylin and eosin (H&E) staining. Morphological examination of tumors showed increased angiogenesis signs surrounding the control-treated mouse tumor surface (Figure 2E, left). Moreover, histological analysis of tumor lesions demonstrated typical TNBC-associated morphological features in the placebo control-treated mouse tumors, including a ribbon-like architecture related to central necrosis, increased geographic tumor necrosis, and apoptotic body and stromal lymphocytic response (Figure 2E, left and Supplementary Figure S1) [32,38]. In contrast, these advanced tumor features could not be detected in OC-X treated mice, which only had typical malignancy features represented by solid sheets of malignant cells. On the other hand, control group mouse lungs demonstrated loss of the highly vascular flat shiny surface compared to the OC-X treated mouse lungs, which contrarily showed well-structured and less vascular morphology (Figure 2E, right). H&E stained lung section histopathological analysis further indicated that the control group mouse lungs were occupied by multiple lesions with several nuclei, while few such lesions were obviously visible in the OC-X treated mice group.

### 3.2. Antitumor Activity of OC-X in the PDX Animal Model

**Figure 3:**
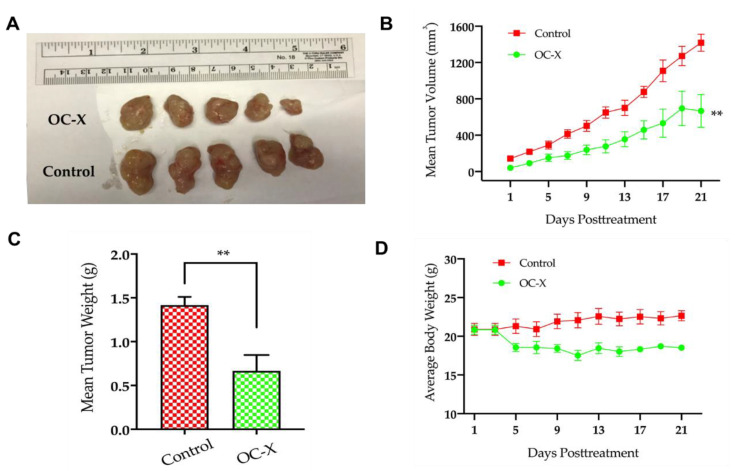
OC-X suppressed tumor growth in PDX mice. (**A**) Comparison of representative tumor images of the OC-X treatment versus the control group PDX mice (*n* = 5, each). (**B**) Tumor volumes were monitored for OC-X treated and control groups and measured over the 21 days of the course of the experiment. Tumor volume (V) was calculated using the formula V = L/2 × W2. (**C**) Comparison of collective mean tumor weight collected from the glands of treatment and control groups after mice were sacrificed at the experiment end. (**D**) The average body weights for each mice group were monitored over the experiment duration. No significant mice body weight change was observed between treated and control animals, regardless of the slight weight decrease for treated mice in the initial treatment days. The results are expressed as the mean SEM; *p*-value indicated by using Student’s *t*-test, ** *p* < 0.01.

The in vivo efficacy of OC-X to suppress human patient heterogeneous TNBC patient-derived tumor xenografts (PDXs) implanted in severely combined immunodeficient (SCID) mice was assessed (Figure 1). The PDX mouse model was essentially established by transplanting a segment of a human patient tumor into SCID mice, which maintains the genomic preservation of their human primary tumors over multiple serial passages [39]. PDX mice reflect the major characteristics of the clinical data of original human tumors, including cellular heterogeneity and microenvironment interfering, genomic profiles, and treatment receptivity [40,41]. The Jackson Laboratory PDX mice TM00089 were treated by oral gavage with OC-X (equivalent 7.5 mg/kg OC), *n* = 5, or placebo control, *n* = 5, 6×/week for approximately three consecutive weeks. The results demonstrated a significant reduction in tumor volume and tumor weight compared to the vehicle-treated control group (Figure 3A). OC-X treatments suppressed 47% of tumor growth at the study end versus the vehicle-treated control group. The mean mice tumor volume on the final study day was 1417 ± 94 mm^3^ and 666 ± 180 mm^3^ for the vehicle control and OC-X treated groups, respectively (Figure 3B). Furthermore, the mean tumor weight in the vehicle-treated control group was 1.91 ± 0.17 g, while the mean tumor weight for the OC-X treated mice group was 1.09 ± 0.22 g (Figure 3C). OC-X treatment caused a mild reduction in mice body weight at the initial treatment days, but, later, this weight reduction was stabilized for the rest of the study course, and did not cause any additional animal weight reduction versus the placebo group, suggesting possible preliminary safety (Figure 3D).

### 3.3. Microarray-Based Gene Signature of OC-X Treatments in MMTV-PyVT and PDX Mouse Tumors

**Figure 4:**
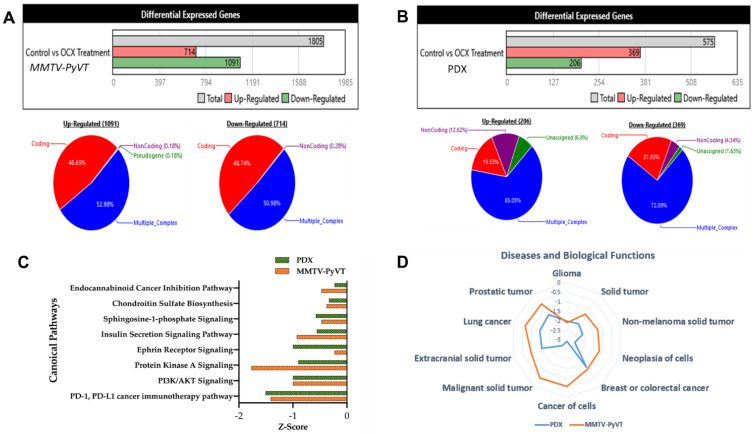
Comparative microarray analysis of OC-X treatment effects in MMTV-PyVT transgenic and PDX mice tumors using Mice and Human Clariom S arrays, respectively, highlighted the differential gene expression and ontology (GO) results. Transcriptome analysis using the Affymetrix Transcriptome Analysis Console (TAC) filtered and assessed all 1.8-fold expression dysregulations with nominal significance, and then characterized all genes affected by OC-X treatments into up- and downregulated based on their global dataset expression in quantitative schematic representations. (**A**) MMTV-PyVT mouse tumor array analysis. (**B**) PDX mouse tumor array analysis. The majority of deregulated genes were categorized as coding and multiple complex genes, the most interrogated category. (**C**) Ingenuity pathway analysis (IPA) revealed the top downregulated canonical pathways, along with predicted activation scores. (**D**) OC-X treatments predicted the affected cellular functions and diseases with the lowest z-scores. Analyses focused on the cancer-related and overlapping pathways in both mouse models.

Tumor samples of OC-X and control-treated MMTV-PyVT and PDX mice were subjected to Mouse/Human Gene Chip Clariom™ S array analysis, which serves as a next-generation transcriptome profiling tool. This technology is widely used for the detection of differentially expressed genes (DEGs) to explore gene-level expression correlation among treatment groups, revealing the effectiveness of the targeted treatment [33]. Preliminary bioinformatics analysis of the raw data of the MMTV-PyVT tumor sample results of the Mouse Clariom S Microarray analysis revealed 1805 genes affected by OC-X treatment out of 22,206 genes (Figure 4A). Among these, 714 genes were upregulated and 1091 genes were downregulated. About 52.98% of the upregulated genes originated from multiple complexes, while 46.65% were coding genes (Figure 4A, left circle). Nearly 50.98% of the downregulated genes originated from multiple complexes, and 48.74% were coding genes (Figure 4A, right circle). On the other hand, microarray analysis of the PDX mice tumor samples preformed using the Human Clariom S Microarray analysis showed a total of 575 differentially expressed genes affected by OC-X treatment out of 21,448 genes (Figure 4B). Among them, 369 genes were upregulated and 206 genes were downregulated. The upregulated genes contained 65.05% that originated from multiple complexes, while 15.53% were coding genes (Figure 4B, left circle). The downregulated genes contained 72.09% that originated from multiple complexes, while 21.95% were coding genes (Figure 4B, right circle).

### 3.4. Predicted and Overlapped Canonical Pathways and Presumptive Diseases in Response to OC-X in MMTV-PyVT and PDX Mouse Tumors

Ingenuity pathway analysis (IPA) software evaluated the canonical pathways affected by OC-X treatments in MMTV-PyVT and PDX mouse tumors, offering insights into gene-level signatures in these heterogeneous TNBC representative models. Canonical pathways analysis took advantage of the IPA library to predict the markedly affected signaling pathways associated with the most differentially expressed genes in response to OC-X treatments [42]. Affected canonical pathways evaluated in both animal models were based on the z-value, which compares the significance of the affected pathways between treatment and control datasets. A negative z-score indicates deactivation of a particular pathway functionality compared to the range of normal activity in the database. One striking outcome of the IPA predictive analysis was highlighting the overlapped top-most downregulated PD-1/PD-L1 cancer immunotherapy pathway, PI3K-AKT, and protein kinase A signaling pathways in both MMTV-PyVT and PDX mouse tumors following OC-X treatments versus the control group (Figure 4C). In addition, Figure 4D graphically represents the most relevant cancer-related diseases affected by OC-X treatments in MMTV-PyVT and PDX datasets. The IPA database suggested the potential suppression of glioma, non-melanoma, breast, colorectal, lung, and prostate cancers by 1–3-fold toward a negative z-score. Comprehensively, there are also other non-overlapping canonical pathways and predicted diseases individually affected in either mouse model with clear biological relevance (Supplementary Figure S2A,B).

### 3.5. Pathway Analysis Gives Insight into Gene Differentiation Expressed in MMTV-PyVT and PDX Array Data after OC-X Treatment

IPA was used to determine the OC-X treatment effects on the expression levels of genes regulating different TNBC pathogenesis stages, including neoplasia, cell-to-cell adhesion signaling and interaction, invasion, migration, and metastasis in MMTV-PyVT and PDX mouse tumors based on the z-value, with a two-fold cutoff. The results showed that multiple genes affected either upregulation or downregulation in each stage, including some overlapping affected genes in both mouse models. The study focused only on the cancer pathogenesis-associated genes affected by OC-X treatments through relying primarily on the IPA database diseases and function prediction tool.

**Figure 5:**
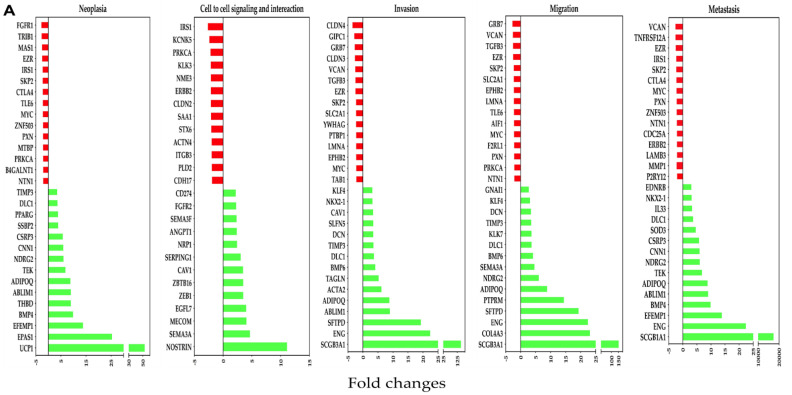

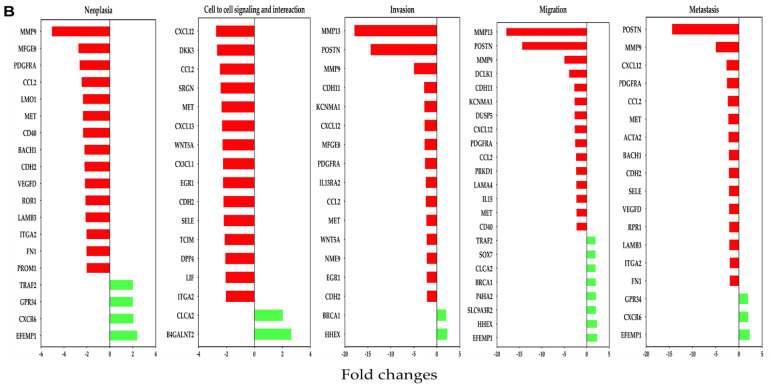
Association of differentially affected genes by OC-X treatments with TNBC progression stages (neoplasia, cell-to-cell adhesion signaling and interaction, invasion, migration, and metastasis) in MMTV-PyVT and PDX mouse tumors. (**A**) Affected genes in MMTV-PyVT transgenic mouse tumors. (**B**) Affected genes in PDX mouse tumors. IPA regulator and functional prediction analysis was based on negative (red bars) and positive (green bars) z-scores. Some common genes contribute to multiple TNBC progression stages due to its relevance in TNBC pathogenesis.

Analysis of genes downregulated by OC-X treatments in the tumor originally generated in MMTV-PyVT mice mammary glands revealed multiple affected genes with a marginal alteration in the z-score. Affected genes included FGFR1, IRS1, CLDN4, GRB7, and VCAN, while EZR and MYC were commonly affected in at least four out of five progression stages of the selected cancer levels. OC-X treatments also caused surge upregulation of several important biomarkers within each cancer progression stage, including UCP1, NOSTRIN, SCGB3A1, ENG, EPAS1, SFTPD, and BMP4 (Figure 5A).

In the PDX model, most downregulated genes affected by OC-X treatments included MMP9, CXCL12, MMP13, and POSTN within the selected cancer stages. Moreover, several other commonly important genes were also reduced by more than two-fold, including CCL2, PDGFRA, MET, ROR1, and FN1. On the other hand, EFEMP1, B4GALNT2, and HHEX were predicted to be the topmost upregulated genes, with the highest z-values in the human PDX tumors (Figure 5B).

### 3.6. Comparison of OC-X Treatment Genes Signature and Transcriptomic Profiles Identified Affected Overlapping Genes Within MMTV-PyVT and PDX Mouse Tumors

**Figure 6:**
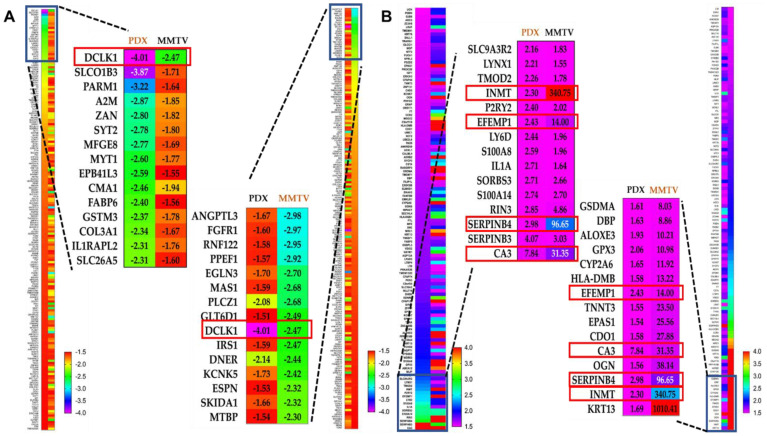
Comparative ingenuity pathway analysis (IPA) of the differential expression of genes affected in both MMTV-PyVT and PDX mice tumor arrays. (**A**) The heat map of the topmost downregulated genes overlapped in both MMTV-PyVT and PDX models. (**B**) The heat map visualization of the topmost upregulated genes overlapped in both MMTV-PyVT and PDX models. The magnified part visualizes the quantitative topmost 15 genes within each dataset. Left magnification panel; topmost PDX mouse tumor genes arranged in ascending order compared with their counterparts in MMTV-PyVT mouse tumors. Right magnification panel; topmost MMTV-PyVT mouse tumor genes arranged in ascending order compared with their counterparts in PDX mouse tumors. The complete overlapped genes list is provided in Supplementary Material.

Comparing and matching the mice and human tumor microarray data is an exemplary approach for preclinical validation and better understanding OC molecular mechanisms and pharmacology. To test the overall similarity between OC-X treatment effects on gene expression alteration in MMTV-PyVT and PDX mouse tumors, the predicted pathway activity generated from each model tumor was compared using the IPA comparison analysis tool. This IPA modality graphically depicts the expected similarity in pathway activity based on the observed overlap in up- and downstream gene expression. Without applying any filtration cutoffs, about 5849 genes were affected by OC-X treatments in MMTV-PyVT compared to 3085 genes affected in PDX. However, a filtration cutoff of 1.5-fold of the z-score was applied, along with the elimination of the less relevant heat map biomarkers, as no analysis satisfied the filter cutoff. The resulting heat maps showed over 192 downregulated genes overlapped in both models, with various z negative values (Figure 6). Meanwhile, only 120 overlapping genes were upregulated.

Considering the different origin of array data and the z-score variation between both models within the same gene, the overlapping genes were sorted in an ascending order based on the z-score in the PDX dataset (Figure 6A, left panel), then sorted with the same order, but based on the z-score in the MMTV-PyVT dataset (Figure 6A, right panel). Among the top 15 genes proved common in both models, the comparison analysis revealed DCLK1 as the only downregulated gene with a significant z-score, −2.47-fold and −4.01-fold in MMTV-PyVT and PDX, respectively. Similarly, using the same approach in the topmost upregulated genes, CA3 showed 31.35- and 7.84-fold, SERPINB4 showed 96.65- and 2.98-fold, EFEMP1 showed 14.0- and 2.43-fold, and INMT showed 340.75- and 2.3-fold upregulation in MMTV-PyVT and PDX, respectively (Figure 6B).

**Figure 7:**
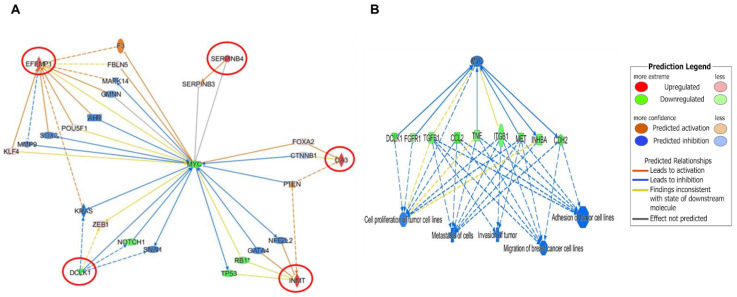
Predicted IPA-generated network mapping of OC-X treatment effects on the signal transduction and molecular interactions of the five topmost affected genes that overlapped in both MMTV-PyVT and PDX array data. (**A**) Predicted mapping of affected genes was connected based on direct (solid lines) and indirect (dotted lines) interactions with MYC and its intermediate regulators, the best gene linked to the affected genes. The saturation of color is directly proportional to each gene fold change. (**B**) IPA-generated predicted correlation of MYC-driven genes and TNBC progression stage in response to OC-X treatment.

The IPA Gene Growing Analysis tool was used to uncover the affected genes associated with DCLK1, CA3, SERPINB4, EFEMP1, and INMT individually and to trace the biological interplay of these genes with other relevant molecular pathways (Supplementary Figure S3). To find potential upstream regulators, which can affect the expression of these genes, the IPA Connection Analysis tool was used to simultaneously connect all affected genes. Interestingly, MYC was identified as an upstream biomarker that could associate directly and indirectly with DCLK1, SERPINB4, and EFEMP1, but could only connect indirectly to INMT and CA3 (Figure 7A). Additional data analyses revealed that the main biological function outcomes of OC-X treatments could potentially be attributed to the MYC downregulation by a more than −2.4-fold change, plausibly suppressing all previously reported TNBC progression stages, including neoplasia, cell proliferation, cell-to-cell signaling and interaction, invasion, migration, and metastasis (Figure 7B). The literature expression profile of MYC in clinical tumor and normal samples was conducted via the UALCAN database analysis tool, which highlighted the clinical significance of MYC across various cancers (Supplementary Figure S4).

## 4. Discussion

Natural products have a standing success record for the control of various cancer types [43,44]. Natural products are gaining increased attention for their ample sustained sources, target novelty and selectivity, plausible relative safety profiles, and cost-effectiveness compared to conventional synthetic drugs [45]. Previous studies on OC, a representative mono-phenolic secoiridoid primarily extracted from EVOO, manifested its inhibitory effects on BC cell proliferation, migration, invasion, and angiogenesis. The OC anti-BC activity margin included TNBC and luminal BC phenotypes [17,18,22,23,35]. Particularly, OC suppressed the c-Met RTK activation as a major regulator of the progression of multiple cancers, including TNBC. Dysregulation of the HGF/c-Met axis in human malignancies activates various downstream effectors and pathways, such as Akt, MAPK, STAT-3, HSP90, ERα, PI3K, and mTOR, resulting in an aggressive profile [17,18,36]. Moreover, OC suppressed its target, c-Met, in in vivo multiple human BC phenotypes xenografted in nude mice and selectively suppressed the malignancy progression at tolerated daily oral doses of 10 mg/kg and 5 mg/kg intraperitoneally 3×/week [17,18,23,35,36]. Although it is clear that OC exerts antitumor activity against multiple cancers, molecular and gene-level effects have not yet been illustrated in detail [35,46].

The results of the current study validated the OC-X oral administration in experimental animals to suppress mammary carcinogenesis in MMTV-PyVT transgenic mice. Moreover, OC-X suppressed the initiation and incidence of new tumor clusters in MMTV-PyVT mice mammary glands, and inhibited the metastases of BC to the lungs compared to the control group. This study’s results provided evidence that OC-X was able to suppress tumor growth and survival using mice engrafted with human patient BC (PDX), an established pseudoclinical animal model that maintains the pathogenesis of human cancer. However, the results show that OC-X treatment was more effective in MMTV-PyVT than PDX-TNBC mice, possibly due to the advanced progression and aggressiveness of the engrafted human patient tumors in PDX mice and the shorter treatment period compared to the MMTV-PyVT transgenic mice model.

Nevertheless, the remarkable anti-tumor activity of OC-X in preclinically relevant heterogeneous TNBC mice models following an oral administration of 7.5 mg/kg OC-X 6×/week for 75 days in MMTV-PyVT and 21 days in PDX mice neither showed frank signs of systemic toxicity nor significant body weight changes. These findings propose the OC-X formula potentiality to exhibit clinically potent anticancer effects without noticeable toxicity, unlike most targeted cancer therapies.

Microarray-based gene transcription/expression profiling is a well-defined robust trait methodology, which can simultaneously detect the expression levels of all genes and measure the differences between tumor and control samples to distinguish the main changes occurring in response to intervention treatments [47]. Fortunately, most of the genes identified in both tumor samples were coding or multiple complex genes (contained more than one locus type or a complex gene family), which facilitated its connection with the IPA database. IPA analysis predicted the modulation of several cancer-related canonical pathways and biological diseases in both MMTV-PyVT and PDX models in response to OC-X treatments. Microarray results indicated that OC-X treatment suppressed the total c-Met expression level in PDX tissues by 2.38-fold versus the vehicle control. Although the total c-Met expression level change by OC-X was limited, there was a significant suppression of several c-Met downstream effectors due to the potential inhibition of the intratumoral activation level c-Met (Y1234/1235) by OC-X treatment, which has already been reported in several studies [18,20,22,23,36].

This study aimed to identify the OC-X treatment gene signature effects and tracing affected cellular pathways in TNBC MMTV-PyVT and PDX mouse models and the associated progression stages, including neoplasia, cell-to-cell adhesion signaling and interaction, invasion, migration, and metastasis. Indeed, utilizing two advanced heterogeneous TNBC mouse models that have been marketed as preclinical/pseudoclinical models with close similarity to human tumors added translational value to the study outcomes.

In detail, the MMTV-PyVT mice tumor gene microarray analysis identified fibroblast growth factor receptor-1 (FGFR1) as the topmost downregulated gene in the neoplasia stage. The FGFR is a tyrosine kinase receptor that acts as an oncogenic driver conducted for several types of tumors, and thus is assumed as a poor prognostic progression marker in female cancer patients [48]. Amplification of the FGFR is common in several malignancies, including TNBC, and leads to the induction of tumor cell proliferation and migration, blockage of apoptosis, and promotion of epithelial-to-mesenchymal (EMT) transitions [49]. Thus, suppression of FGFR1 expression by OC-X treatments was fundamental in reducing the MMTV-PyVT mouse tumor volume. Furthermore, OC-X treatments downregulated the expression of insulin receptor substrate (IRS), another critical cell cycle regulator that has been extensively dysregulated in cancer cells [50]. IRS1 is a cytoplasmic adaptor protein that transmits signals from insulin and insulin-like growth factor 1 (IGF1) to conduct normal cellular and metabolic processes [50]. Cancer cells could dominate the IRS expression level to promote proliferation and survival, along with a response from IGF-1 and its downstream signal transducers, resulting in an elevated expression level of ERα in hormone-dependent BC, which converts to TNBC through the tumor progression sequence [51]. Therefore, OC-X modulation of the IRS signal transduction pathway could provide a gleam of potential to control TNBC and other IRS-dependent cancers. Several other important protein coding genes, such as protein kinase C alpha type (PRKCA), MYC proto-oncogene/BHLH transcription factor (MYC), S-phase kinase associated protein 2 (SKP2), and ezrin (EZR), have also been downregulated two-fold or more by OC-X treatments. Extensive studies in the literature indicate that these genes commonly augment BC and other cancer growth, neoplasia, invasion, migration, and metastasis [52,53,54,55]. The overexpression of these genes in BC correlates with poor patient survival [55]. This finding justifies the OC-X treatment antimetastatic activity in the MMTV-PyVT mice model, which was associated with a significant reduction of these genes’ expressions in the collected tumor samples.

On the other hand, the transcriptome array results strongly indicate that OC-X treatments modulated MMTV-PyVT mice tumor progression, survival, and metastasis through significantly upregulating multiple cancer-related biomarkers and their downstream pathway genes. Microarray results proved that OC-X treatments markedly enhanced mitochondrial uncoupling protein 1 (UCP1), secretoglobin (SCGB) 3A1, nitric oxide synthase traffic inducer (NOSTRIN), and endoglin (ENG) expression levels by at least five-fold or more versus the vehicle control. A brief look at these genes in the literature demonstrates that the overexpression of UCP1 selectively suppressed BC growth by inducing mitochondrial dysfunction, leading to the activation of its cellular catabolism pathway [56]. Furthermore, SCGB3A1 was originally identified in both mouse and human tumor specimens [57]. Evidently, the SCGB3A1 tumor suppressive functions were illustrated as the inhibition of malignant cell growth, invasion, and migration through the AKT signaling pathway [58]. Silencing of SCGB3A1 expression through methylation is well-established in human breast, lung, prostate, and pancreatic cancers [57]. Substantially, this study documented the promoting effect of OC-X treatments on the SCGB3A1 pathway, translated by a more than 17,351-fold upregulation, the highest upregulated gene in the dataset. In contrast to SCGB3A1, the functional role of the endothelial NOSTRIN in cancer has not been fully described, although, in pancreatic cancer, NOSTRIN has been identified as a protein that binds to the endothelial nitric oxide synthase (eNOS) and manipulates its intracellular efficiency, reducing nitric oxide (NO) generation, which directly suppresses the tumor’s migratory and invasive properties [59]. Likewise, another stimulatory effect for OC-X treatment is its promotion of ENG expression. ENG is a protein primarily upregulated during angiogenesis, and can inhibit cell migration, invasion, and metastatic colonization in vitro and in vivo by mitigating the effects of transforming growth factor beta protein (TGF-β), which is activated during the invasion and migration oncogenic stages [60]. Thus, elevated ENG expression is associated with an improved primary tumor response rate for neoadjuvant therapy, which is correlated with improved clinical outcomes in several studies [61].

Similar to the above findings, this study observed a list of important genes related to the neoplasia, cell-to-cell signaling and interaction, invasion, migration, and metastasis effectively manipulated by OC-X treatments in PDX mouse tumors. For instance, IPA gene analyses revealed matrix metallopeptidase 9 (MMP9) as the most downregulated gene in the neoplasia stage. Interestingly, MMP9 has a fundamental role in promoting invasion, migration, and metastasis in breast and several other malignancies. Of note, numerous studies have already shown that MMP9 is an influential prognostic marker for TNBC and HER2 positive BC [62]. Moreover, MMP13 is another affected MMP family gene that shares common structural and functional similarities with MMP9, and is validated to play major roles in TNBC pathogenesis. The major role of MMP overexpression in TNBC is enhancing invasion and subsequent metastasis in patients by degradation of the extracellular matrix (ECM), allowing cancer cells to metastasize freely [63]. Several other important genes were also downregulated by more than two-fold in response to OC-X treatments in PDX mice. These included C-X-C motif chemokine ligand 12 (CXCL12), c-Met, receptor tyrosine kinase-like orphan receptor 1 (ROR1), and fibronectin 1 (FN1), which are associated with activated neoplasia, invasion, migration, and metastasis. In fact, activation of these genes proved to promote TNBC aggressiveness, growth, and metastasis [64,65,66,67].

On the other hand, OC-X treatments showed a marginal upregulating effect on PDX model genes. However, OC-X treatments notably boosted haematopoietically expressed homeobox (HHEX) expressions among the most affected genes with a wide role in TNBC and other cancers. The HHEX is an essential transcription factor that exhibits tumor suppression by regulating the translation of specific mRNAs in various breast, liver, and thyroid cancers [68]. HHEX overexpression can also regulate the transcription of vascular endothelial growth factor (VEGF) and TGFβ co-receptor endoglin, suppressing BC cell proliferation and migration [69]. Clinically, lower HHEX expression is correlated with disease-free survival in BC patients [70]. Low HHEX expression is common in TNBC and hormone-negative and basal-like BCs [70]. Furthermore, β1,4-N-acetylgalactosaminyltransferase 2 (B4GALNT2) is a biosynthetic enzyme of histo-blood group antigens Sda antigen, a natural component of the red blood cells in the healthy colon of a vast majority of people [71]. Prior studies have shown that the downregulation of B4GALNT2 can be a marker for colon and gastrointestinal cancers [71,72]. Direct upregulation of the B4GALNT2 gene level reduces the colon malignancy and the stem-associated malignant phenotype in a highly selective fashion [73]. To our knowledge, this is the first report of systemic B4GALNT2 upregulation in breast cancer, and further investigation is needed to validate the present findings.

The main purpose of this study was to compare the OC-X treatment effects on key genes and mainstream regulators that differentially modulate expressed genes in two heterogeneous TNBC preclinical mouse models. Therefore, we performed pairwise gene code comparisons utilizing IPA analysis to identify the common genes with a significant confidence prediction z-score in both models. The results focused on the topmost affected 15 genes in both MMTV-PyVT and PDX tumor lysate arrays. Therapeutically, the doublecortin-like kinase 1 (DCLK1) was the most useful downregulated gene, while INMT, CA3, Sirpinb4, and EFEMP1 were the best upregulated genes, overlapping in both mouse models. Interestingly, these genes play a crucial role in TNBC and other cancers. DCLK1 is a microtubule binding kinase protein, which has been extensively studied in the field of neurodevelopment due to its microtubule polymerizing function of the doublecortin domains that are crucial for neuronal migration [74]. In the cancer context, DCLK1 plays a role in the cellular migration of multiple types of cancer, including colorectal, pancreatic, liver, and breast cancers [75]. Dysregulation of DCLK1 in humans and mice is associated with activated pathological cancer stages. DCLK1 is a cancer stem cell (CSC) marker, which promotes initiation, aggressiveness, and metastasis through many pathways, such as the Wnt/β-catenin pathway [76,77]. Thus, the OC-X treatment suppression of DCLK1 in both models implies potential therapeutic applications to control TNBC.

The association between CA3, SERPINB4, EFEMP1, and INMT expressions with the TNBC progression remains a matter of debate. For example, carbonic anhydrases 3 (CA3) is a cytoplasmic metalloenzyme protein, whose function is to mediate the aging oxidative insult, and is considered a marker for cancer cell hypoxia [78,79]. However, the roles of CA3 in BC pathogenesis are still unclear [78,79]. Concurrently, the serine/cysteine protease inhibitor SERPINB3, which is directly manipulated by SERPINB4, is modulated by hypoxia through the hypoxia-inducible factor-2α (HIF-2α), and has been recently proven to be upregulated in human hepatoblastoma [79,80]. However, no relevant literature validates the relationship between the expression of SERPINB3 or 4 and clinical outcomes in BC [80,81]. Meanwhile, indolethylamine-*N*-methyltransferase (INMT) is a type 1 transmethylation enzyme known for its capability to deliver methyl groups, forming methylated compounds, such as N,N-dimethyltryptamine (DMT). Reduced expression of INMT has been associated with enhanced non-small cell lung cancer (NSCLC) adenocarcinomas and prostate cancer [82,83]. However, there is no evidence illustrating the role of INMT in breast cancer. Finally, there are conflicting reports regarding the function of EGF-containing fibulin-like extracellular matrix protein 1 (EFEMP1) in diverse cancer types. For example, overexpression of EFEMP1 eliminated tumor development and suppressed angiogenesis and VEGFA expression in hepatocellular carcinoma, gastric and prostate cancers, and glioblastoma [84,85], while the converse was true with ovarian and breast cancers [86]. Overall, our data reveal that OC-X treatment can upregulate these genes confidently in both models, and thus, further study is needed in the future for the effect of each particular gene with OC.

Interestingly, the generation of gene networks for the top five overlapping affected genes demonstrated that MYC can be the only direct or indirect connector for these genes. Thus, the relationship between MYC and these genes should be within the understanding of the essential role of MYC in TNBC progression. MYC is a heterogeneous proto-oncogene encoding various transcription factors, leading to participation in many aspects of tumor cellular functions. Indeed, numerous clinical studies have suggested MYC overexpression as a marker for poor outcomes in TNBC patients through various pathways and mechanisms, including: (i) promoting cell proliferation and survival via PI3K, (ii) mediating mammary stem cell amplification as a cascade of Wnt/β-catenin activation, (iii) accelerating cell cycle activity associated with a high expression of cyclin B1 and Ki67, and (iv) enhancing tumor angiogenesis by activating VEGF signaling [87,88]. Impressively, results showed a 2.4-fold downregulation of MYC in response to OC-X treatments in MMTV-PyVT mice, combined with the effects of its up- and downstream effectors. However, a slight increase in MYC expression was observed in the PDX model. Consistent with the previous OC suppressive effect on c-Met, the result of combining MYC blockade and c-Met inhibition in heterogeneous mouse models heightened the therapeutic potential of OC from various oncological and biological aspects. Finally, modification of MYC by manipulating the five abovementioned genes in both animal models possibly represents a good starting point for better future understanding of OC molecular mechanisms in TNBC.

## 5. Conclusions

In summary, this study demonstrated the OC-X suppressed the progression and initiation of heterogeneous mouse and human TNBC in valid preclinical and pseudo-clinical advanced animal models. The study findings provide preclinical evidence supporting OC-X’s potential as a promising nutraceutical active against heterogeneous TNBC. Differential gene expression analysis of heterogeneous TNBC microarray data was an exemplary approach for improving the understanding of the molecular effects, gene signature, and pharmacodynamics of OC-X, which may aid the prediction of new molecular target pathways. Comparing the microarray data from mice and human tumor origins proved a promising strategy to assess and validate the clinical validity in human subjects and identify the most affected genes in response to the OC-X treatments. MYC was identified as a key driver for the in vivo anti-BC effects of OC. Based on these results, OC-X assumed as a potential nutraceutical formulation useful for translational application to control TNBC.

## Supplementary Materials

The following are available online at https://www.mdpi.com/article/10.3390/nu13051706/s1, Figure S1: Magnification of the observed H&E staining tumor micromorphology changes in MMTV-PyVT control tumors, Figure S2: Ingenuity pathway analysis (IPA) summary reports for MMTV-PyVT and PDX tumor samples. Figure S3: Predicted mapping of genes to be affected by OC treatment for the top overlapped genes in MMTV-PyVT and PDX datasets. Figure S4: Expression of MYC in different human samples.
