# Supplementary material for: Comparative Gene Signature of (−)-Oleocanthal Formulation Treatments in Heterogeneous Triple Negative Breast Tumor Models: Oncological Therapeutic Target Insights
Source: Nutrients. 2021 May 18;13(5):1706. doi: 10.3390/nu13051706 (PMC8157589; doi:10.3390/nu13051706)
Supplement: Supplementary file 1 [file nutrients-13-01706-s001.zip › nutrients-1200884-supplementary.pdf]

nutrients

Article

Comparative Gene Signature of (−)-Oleocanthal Formulation Treatments in Heterogeneous Triple Negative Breast Tumor Models: Oncological Therapeutic Target Insights

Mohammed H. Qusa, Khaldoun S. Abdelwahed, Abu Bakar Siddique, Khalid A. El Sayed *

School of Basic Pharmaceutical and Toxicological Sciences, College of Pharmacy, University of Louisiana at Monroe, 1800 Bienville Drive, Monroe, LA 71201, USA; qusamh@warhawks.ulm.edu (M.H.Q); abdelwks@warhawks.ulm.edu (K.S.A); siddiqab@warhawks.ulm.edu (A.B.S.)
* Correspondence: elsayed@ulm.edu; Tel.: +1-318-342-1725

Citation: Qusa, M.H.; Abdelwahed, K.S.; Siddique, A.B.; El Sayed, K.A. Comparative Gene Signature of (−)-Oleocanthal Formulation Treatments in Heterogeneous Triple Negative Breast Tumor Models: Oncological Therapeutic Target Insights. *Nutrients* **2021**, *13*, 1706. https://doi.org/10.3390/nu13051706

Received: date
Accepted: date
Published: date

Publisher's Note: MDPI stays neutral with regard to jurisdictional claims in published maps and institutional affiliations.

Copyright: © 2021 by the authors. Submitted for possible open access publication under the terms and conditions of the Creative Commons Attribution (CC BY) license (http://creativecommons.org/licenses/by/4.0/).

Supplementary Materials:

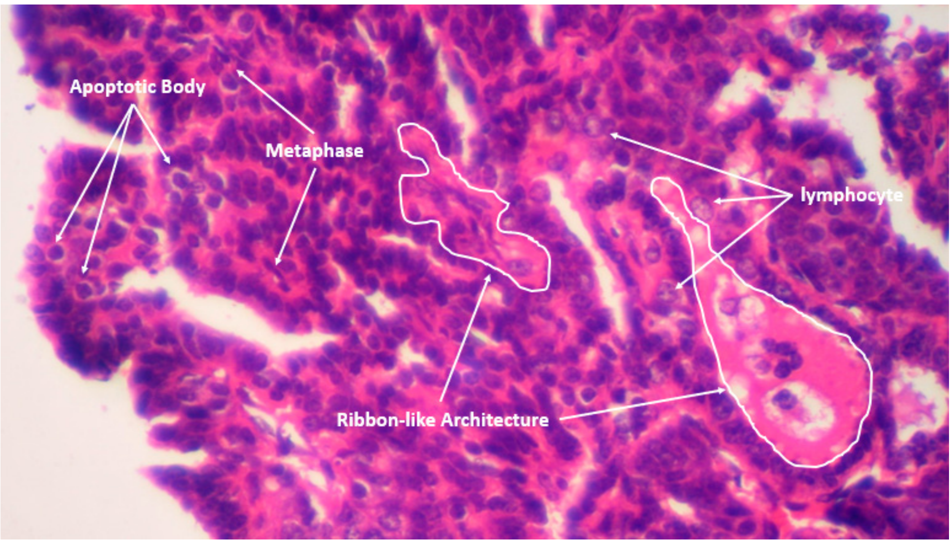

Supplementary Figure S1: Expansion of the H&E stained tumor micromorphology changes in MMTV-PyVT control tumor.

A

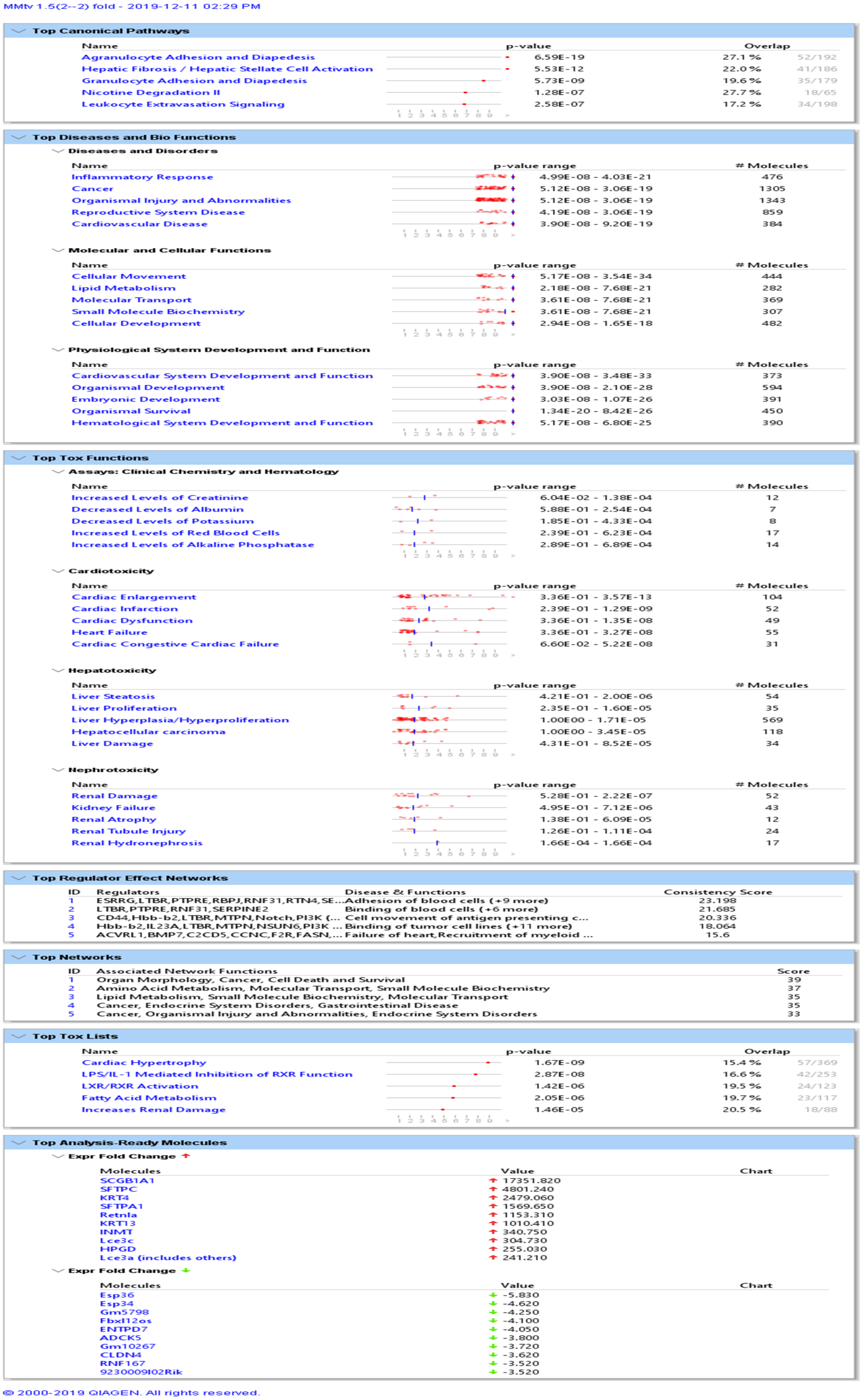
MMtv 1.5(2--2) fold - 2019-12-11 02:29 PM
Top Canonical Pathways
Name p-value Overlap
Agranulocyte Adhesion and Diapedesis 6.59E-19 27.1 % 52/192
Hepatic Fibrosis / Hepatic Stellate Cell Activation 5.53E-12 22.0 % 41/186
Granulocyte Adhesion and Diapedesis 5.73E-09 19.6 % 35/179
Nicotine Degradation II 1.28E-07 27.7 % 18/65
Leukocyte Extravasation Signaling 2.58E-07 17.2 % 34/198
Top Diseases and Bio Functions
Diseases and Disorders
Name p-value range # Molecules
Inflammatory Response 4.99E-08 - 4.03E-21 476
Cancer 5.12E-08 - 3.06E-19 1305
Organismal Injury and Abnormalities 5.12E-08 - 3.06E-19 1343
Reproductive System Disease 4.19E-08 - 3.06E-19 859
Cardiovascular Disease 3.90E-08 - 9.20E-19 384
Molecular and Cellular Functions
Name p-value range # Molecules
Cellular Movement 5.17E-08 - 3.54E-34 444
Lipid Metabolism 2.18E-08 - 7.68E-21 282
Molecular Transport 3.61E-08 - 7.68E-21 369
Small Molecule Biochemistry 3.61E-08 - 7.68E-21 307
Cellular Development 2.94E-08 - 1.65E-18 482
Physiological System Development and Function
Name p-value range # Molecules
Cardiovascular System Development and Function 3.90E-08 - 3.48E-33 373
Organismal Development 3.90E-08 - 2.10E-28 594
Embryonic Development 3.03E-08 - 1.07E-26 391
Organismal Survival 1.34E-20 - 8.42E-26 450
Hematological System Development and Function 5.17E-08 - 6.80E-25 390
Top Tox Functions
Assays: Clinical Chemistry and Hematology
Name p-value range # Molecules
Increased Levels of Creatinine 6.04E-02 - 1.38E-04 12
Decreased Levels of Albumin 5.88E-01 - 2.54E-04 7
Decreased Levels of Potassium 1.85E-01 - 4.33E-04 8
Increased Levels of Red Blood Cells 2.39E-01 - 6.23E-04 17
Increased Levels of Alkaline Phosphatase 2.89E-01 - 6.89E-04 14
Cardiotoxicity
Name p-value range # Molecules
Cardiac Enlargement 3.36E-01 - 3.57E-13 104
Cardiac Infarction 2.39E-01 - 1.29E-09 52
Cardiac Dysfunction 3.36E-01 - 1.35E-08 49
Heart Failure 3.36E-01 - 3.27E-08 55
Cardiac Congestive Cardiac Failure 6.60E-02 - 5.22E-08 31
Hepatotoxicity
Name p-value range # Molecules
Liver Steatosis 4.21E-01 - 2.00E-06 54
Liver Proliferation 2.35E-01 - 1.60E-05 35
Liver Hyperplasia/Hyperproliferation 1.00E00 - 1.71E-05 569
Hepatocellular carcinoma 1.00E00 - 3.45E-05 118
Liver Damage 4.31E-01 - 8.52E-05 34
Nephrotoxicity
Name p-value range # Molecules
Renal Damage 5.28E-01 - 2.22E-07 52
Kidney Failure 4.95E-01 - 7.12E-06 43
Renal Atrophy 1.38E-01 - 6.09E-05 12
Renal Tubule Injury 1.26E-01 - 1.11E-04 24
Renal Hydronephrosis 1.66E-04 - 1.66E-04 17
Top Regulator Effect Networks
ID Regulators Disease & Functions Consistency Score
1 ESRRG,LTBR,PTPRE,RBPJ,RNF31,RTN4,SE... Adhesion of blood cells (+9 more) 23.198
2 LTBR,PTPRE,RNF31,SERPINE2 Binding of blood cells (+6 more) 21.685
3 CD44,Hbb-b2,LTBR,MTPN,Notch,PI3K (... Cell movement of antigen presenting c... 20.336
4 Hbb-b2,IL23A,LTBR,MTPN,NSUN6,PI3K ... Binding of tumor cell lines (+11 more) 18.064
5 ACVRL1,BMP7,C2CD5,CCNC,F2R,FASN,... Failure of heart,Recruitment of myeloid ... 15.6
Top Networks
ID Associated Network Functions Score
1 Organ Morphology, Cancer, Cell Death and Survival 39
2 Amino Acid Metabolism, Molecular Transport, Small Molecule Biochemistry 37
3 Lipid Metabolism, Small Molecule Biochemistry, Molecular Transport 35
4 Cancer, Endocrine System Disorders, Gastrointestinal Disease 35
5 Cancer, Organismal Injury and Abnormalities, Endocrine System Disorders 33
Top Tox Lists
Name p-value Overlap
Cardiac Hypertrophy 1.67E-09 15.4 % 57/369
LPS/IL-1 Mediated Inhibition of RXR Function 2.87E-08 16.6 % 42/253
LXR/RXR Activation 1.42E-06 19.5 % 24/123
Fatty Acid Metabolism 2.05E-06 19.7 % 23/117
Increases Renal Damage 1.46E-05 20.5 % 18/88
Top Analysis-Ready Molecules
Expr Fold Change
Molecules Value Chart
SCGB1A1 17351.820
SFTPC 4801.240
KRT4 2479.060
SFTPA1 1569.650
Retnla 1153.310
KRT13 1010.410
INMT 340.750
Lce3c 304.730
HPGD 255.030
Lce3a (includes others) 241.210
Expr Fold Change
Molecules Value Chart
Esp36 -5.830
Esp34 -4.620
Gm5798 -4.250
Fbxl12os -4.100
ENTPD7 -4.050
ADCK5 -3.800
Gm10267 -3.720
CLDN4 -3.620
RNF167 -3.520
9230009I02Rik -3.520
© 2000-2019 QIAGEN. All rights reserved.

B

PDX 1.5 fold(2--2) - 2019-12-11 02:11 PM

Top Canonical Pathways

Name	p-value	Overlap	
Hepatic Fibrosis / Hepatic Stellate Cell Activation	6.90E-09	10.8 %	20/186
Agranulocyte Adhesion and Diapedesis	1.19E-08	10.4 %	20/192
Atherosclerosis Signaling	7.00E-07	11.3 %	14/124
Leukocyte Extravasation Signaling	9.80E-06	8.1 %	16/198
Granulocyte Adhesion and Diapedesis	1.20E-05	8.4 %	15/179

Top Upstream Regulators

Upstream Regulators

Name	p-value	Predicted Activation
Alpha catenin	2.22E-09	Activated
STAT3	2.97E-09	Inhibited
TGFB1	4.13E-09	Inhibited
IL1A	7.92E-09	Inhibited
lipopolysaccharide	1.43E-08	Inhibited

Causal Network

Name	p-value	Predicted Activation
Glucocorticoid-GCR	6.34E-13	
sulindac	7.41E-13	
TAZ	1.14E-12	Inhibited
FHL2	2.91E-12	
Alpha catenin	1.19E-11	Activated

Top Diseases and Bio Functions

Diseases and Disorders

Name	p-value range	# Molecules
Cancer	1.11E-03 - 3.86E-23	474
Dermatological Diseases and Conditions	1.14E-03 - 3.86E-23	340
Organismal Injury and Abnormalities	1.23E-03 - 3.86E-23	478
Reproductive System Disease	1.17E-03 - 5.09E-15	327
Gastrointestinal Disease	1.17E-03 - 1.56E-10	425

Molecular and Cellular Functions

Name	p-value range	# Molecules
Cellular Movement	1.14E-03 - 1.57E-07	134
Cell-To-Cell Signaling and Interaction	1.17E-03 - 1.17E-06	114
Cellular Function and Maintenance	9.71E-04 - 3.48E-06	91
Cellular Growth and Proliferation	1.02E-03 - 5.90E-06	89
Cell Death and Survival	1.02E-03 - 8.70E-06	149

Physiological System Development and Function

Name	p-value range	# Molecules
Tissue Development	1.20E-03 - 8.68E-10	110
Cardiovascular System Development and Function	1.23E-03 - 6.93E-08	99
Organismal Development	1.23E-03 - 2.76E-07	148
Nervous System Development and Function	1.17E-03 - 1.84E-06	89
Hematological System Development and Function	1.05E-03 - 2.23E-06	100

Top Tox Functions

Assays: Clinical Chemistry and Hematology

Name	p-value range	# Molecules
Increased Levels of LDH	5.38E-02 - 5.38E-02	2
Increased Levels of Albumin	2.01E-01 - 8.51E-02	2
Decreased Levels of Albumin	2.53E-01 - 1.26E-01	2
Increased Levels of ALT	1.26E-01 - 1.26E-01	1
Increased Levels of AST	1.26E-01 - 1.26E-01	1

Cardiotoxicity

Name	p-value range	# Molecules
Cardiac Enlargement	3.10E-01 - 3.47E-04	33
Heart Failure	2.70E-01 - 1.80E-03	12
Cardiac Arythmia	5.40E-01 - 2.03E-03	16
Cardiac Damage	2.37E-01 - 4.07E-03	6
Cardiac Degeneration	2.22E-02 - 4.07E-03	4

Hepatotoxicity

Name	p-value range	# Molecules
Liver Hyperplasia/Hyperproliferation	1.00E00 - 4.53E-06	219
Liver Damage	1.90E-01 - 6.81E-04	15
Liver Fibrosis	4.04E-01 - 1.63E-03	9
Liver Cirrhosis	1.26E-01 - 7.76E-03	15
Hepatocellular carcinoma	1.00E00 - 1.39E-02	33

Nephrotoxicity

Name	p-value range	# Molecules
Renal Damage	2.86E-01 - 1.76E-02	6
Renal Tubule Injury	2.86E-01 - 1.76E-02	6
Renal Dilation	2.22E-02 - 1.97E-02	2
Glomerular Injury	5.55E-01 - 2.22E-02	17
Renal Degeneration	2.22E-02 - 2.22E-02	1

Top Regulator Effect Networks

ID	Regulators	Disease & Functions	Consistency Score
1	AGT,AKT1,Ap1,C5,CD40,CD44,cytokine,EDN1,F2,F2R...	Activation of macrophages (+13 more)	59.286
2	AGT,AKT1,IL18,ITGB1,Jnk,MYD88,NOTCH3,P38 MAP...	Activation of macrophages (+6 more)	33.45
3	C5,EGLN,IL17A,MYD88,TICAM1,TLR3,TLR4	Adhesion of tumor cell lines (+3 more)	25.927
4	ETS2,F2R,MYD88,P38 MAPK,SP1,TICAM1,TRADD	Activation of macrophages (+4 more)	22.841
5	F2R,IL18,MYD88,P38 MAPK,PLK4,SEMA7A,TICAM1,...	Activation of macrophages (+5 more)	21.934

Top Networks

ID	Associated Network Functions	Score
1	Cardiovascular Disease, Organismal Injury and Abnormalities, Cell-To-Cell Signaling and Interaction	33
2	Cellular Assembly and Organization, DNA Replication, Recombination, and Repair, Cell Cycle	33
3	Tissue Development, Cancer, Organismal Injury and Abnormalities	31
4	Cellular Movement, Lipid Metabolism, Nervous System Development and Function	31
5	Cancer, Organismal Injury and Abnormalities, Cellular Movement	29

Top Tox Lists

Name	p-value	Overlap	
Genes associated with Chronic Allograft Nephropathy (Human)	4.79E-06	28.6 %	6/21
Acute Renal Failure Panel (Rat)	6.65E-05	12.9 %	8/62
LPS/IL-1 Mediated Inhibition of RXR Function	1.87E-04	6.3 %	16/253
Hepatic Fibrosis	1.87E-04	9.0 %	10/111
Cardiac Hypertrophy	1.66E-03	4.9 %	18/369

Top Analysis-Ready Molecules

Expr Fold Change ↑

Molecules	Value	Chart
CA3	↑ 7.840	
EPGN	↑ 4.430	
S100A2	↑ 4.080	
SERPINB3	↑ 4.070	
POTEB/POTEB2	↑ 3.230	
GREM1	↑ 3.160	
XRCC3	↑ 3.060	
CWH43	↑ 3.020	
SERPINB4	↑ 2.980	
HS3ST3A1	↑ 2.960	

Expr Fold Change ↓

Molecules	Value	Chart
MMP13	↓ -18.020	
POSTN	↓ -14.480	
ACTG2	↓ -7.340	
SPARCL1	↓ -5.630	
NFASC	↓ -5.410	
MMP9	↓ -5.080	
TDRD12	↓ -4.900	
CDR1	↓ -4.550	
GUCY1A1	↓ -4.520	
ODAM	↓ -4.500	

© 2000-2019 QIAGEN. All rights reserved.

Supplementary Figure S2: Ingenuity Pathway Analysis (IPA)-derived summary reports for MMTV-PyVT transgenic (**A**) and PDX mouse tumors (**B**) microarray data.

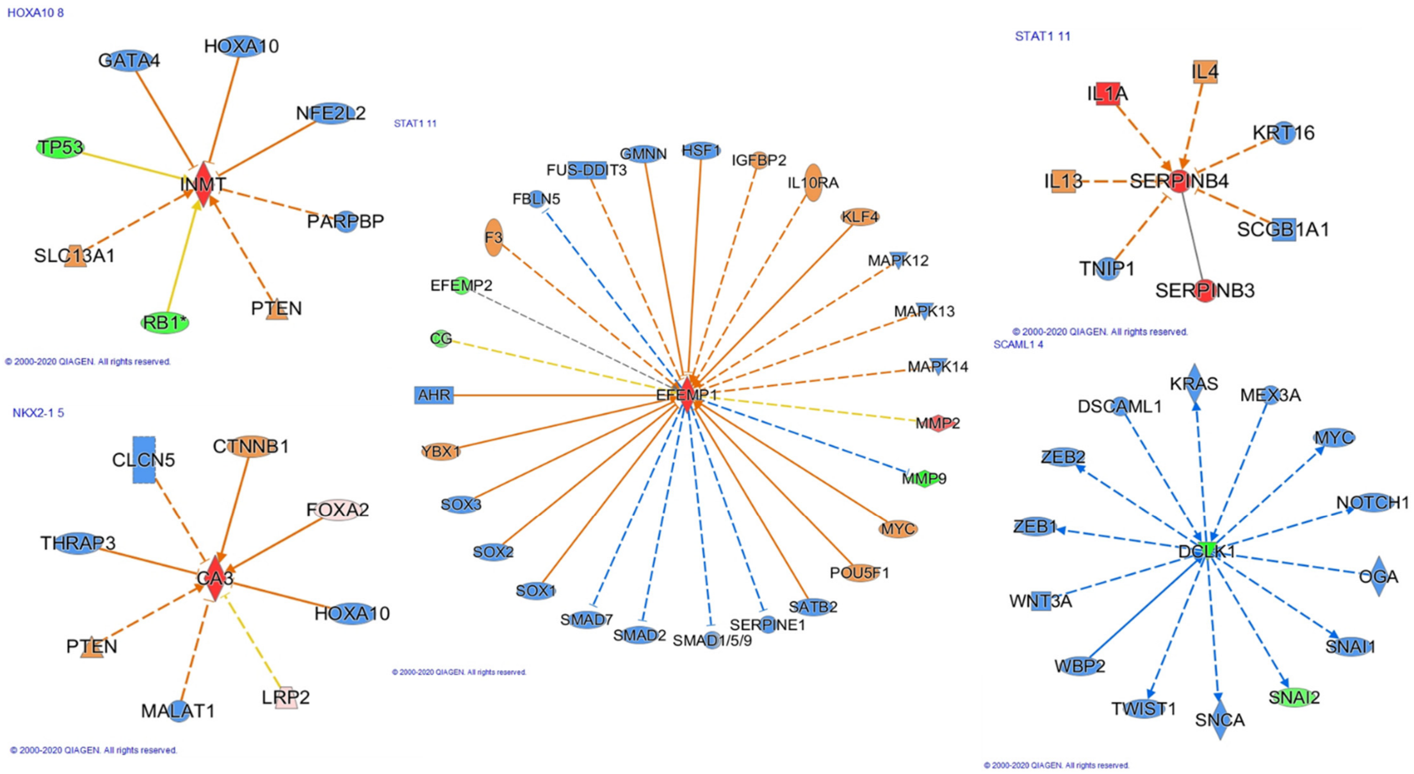

Supplementary Figure S3: IPA-Predicted mapping of the top affected genes by OC-X treatments overlapped in MMTV-PyVT and PDX mice tumor datasets.

A

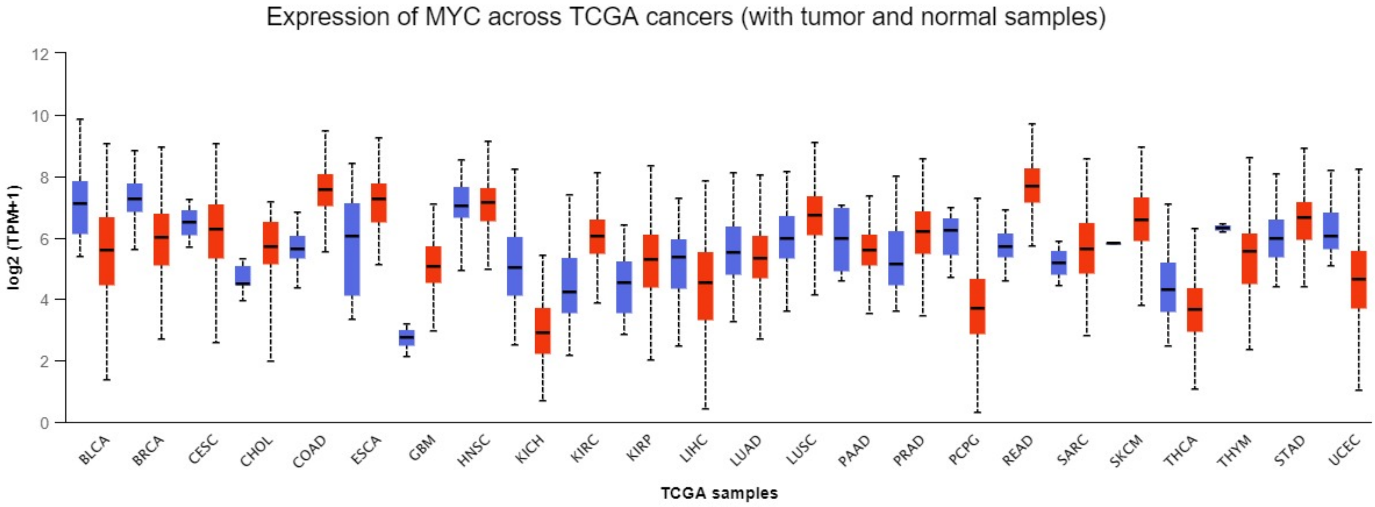

B

Cancer	TCGA code
Bladder urothelial carcinoma	BLCA
Breast invasive carcinoma	BRCA

Cervical squamous cell carcinoma	CESC
Cholangiocarcinoma	CHOL
Colon adenocarcinoma	COAD
Esophageal carcinoma	ESCA
Glioblastoma multiforme	GBM
Head and neck squamous cell carcinoma	HNSC
Kidney choromophobe	KICH
Kidney renal clear cell carcinoma	KIRC
Kidney renal papillary cell carcinoma	KIRP
Liver hepatocellular carcinoma	LIHC
Lung adenocarcinoma	LUAD
Lung squamous cell carcinoma	LUSC
Pancreatic adenocarcinoma	PAAD
Prostate adenocarcinoma	PRAD
Pheochromocytoma and paraganglioma	PCPG
Rectum adenocarcinoma	READ
Sarcoma	SARC
Skin cutaneous melanoma	SKCM
Thyroid carcinoma	THCA
Thymoma	THYM
Stomach adenocarcinomna	STAD
Uterine corpus endometrial carcinoma	UCEC

Supplementary Figure S4: Expression of MYC in different human samples. (**A**) The UALCAN for Cancer Genomics database analysis (http://ualcan.path.uab.edu/cgi-bin/Pan-cancer.pl?genenam=MYC) indicates the predominance of the expression of the mRNAs for MYC within the tumor and normal samples across The Cancer Genome Atlas (TCGA) database. (**B**) Key legend for the TCGA tumor samples.
